# Association of Combined Tobacco Smoking and Oral Contraceptive Use With Cervical Intraepithelial Neoplasia 2 or 3 in Korean Women

**DOI:** 10.2188/jea.JE20150047

**Published:** 2016-01-05

**Authors:** Hea Young Oh, Mi Kyung Kim, Sang-Soo Seo, Jae-Kwan Lee

**Affiliations:** 1 Division of Cancer Epidemiology and Prevention, National Cancer Center, Goyang-si, Korea; 2 Center for Uterine Cancer, National Cancer Center, Goyang-si, Korea; 3 Department of Obstetrics and Gynecology, Korea University College of Medicine, Seoul, Korea

**Keywords:** cervical intraepithelial neoplasia, smoking, oral contraceptive, additive interaction

## Abstract

**Background:**

Cigarette smoking and oral contraceptive (OC) use have been associated with cervical neoplasia, and the combination of smoking and OC use could influence cervical carcinogenesis. We aimed to assess the joint effect of smoking and OC use on the risk of cervical intraepithelial neoplasia (CIN).

**Methods:**

From a cohort of human papillomavirus-positive subjects recruited from 6 hospitals in Korea from March 2006 to November 2012, a total of 678 subjects (411 control, 133 CIN 1, and 134 CIN 2 or 3 cases) were selected for this study (mean age, 43 years). The risk of CIN associated with smoking and OC use on additive and multiplicative scales was estimated via multinomial logistic regression after adjustment for potential confounding factors. The relative excess risk due to interaction (RERI) and the synergy index (S) were used to evaluate the additive interaction.

**Results:**

OC users (odds ratio [OR] 1.98; 95% confidence interval [CI], 1.07–3.69) and long-term OC use (≥20 months; OR 2.71; 95% CI, 1.11–6.59) had a higher risk of CIN 2/3, but had no association with CIN 1, compared to non-OC users. Smokers and heavy smoking (≥8 cigarettes/day) were not associated with any CIN grade. Combined smoking and OC use (OR 4.91; 95% CI, 1.68–14.4; RERI/S, 3.77/27.4; *P* for multiplicative interaction = 0.003) and combined heavy smoking and long-term OC use (OR 11.5; 95% CI, 1.88–70.4; RERI/S, 9.93/18.8; *P* for multiplicative interaction = 0.009) had a higher risk of CIN 2/3 but had no association with CIN 1 compared to combined non-smoking and non-OC use.

**Conclusions:**

OC use and smoking acted synergistically to increase the risk of CIN 2 or 3 in Korean women.

## INTRODUCTION

Oncogenic human papillomavirus (HPV) is a major risk factor of cervical cancer, but cofactors, such as cigarette smoking, long-term oral contraceptive (OC) use, high parity, and co-infection with human immunodeficiency virus, *Chlamydia trachomatis*, or herpes simplex virus type 2, also have been established as risk factors in cervical carcinogenesis.^1^ Smoking and OC use are the most widely studied epidemiological co-factors in cervical cancer development. The International Agency for Research on Cancer (IARC) has determined that tobacco smoking is causally associated with cervical cancer.^2^ In a prospective study including 1800 women with oncogenic HPV DNA, a high smoking intensity among current smokers was associated with an increased risk of CIN 3 or cervical cancer.^3^ The International Collaboration of Epidemiological Studies of Cervical Cancer evaluated the risk of cigarette smoking and found that current smoking increased the risk of cervical squamous cell carcinoma, but not that of adenocarcinoma.^4^^,^^5^ The risk increased up to 3-fold with increasing number of cigarettes smoked per day and years smoked. The risk of cervical cancer among OC users has been long debated.^6^^–^^9^ However, the IARC reported that cervical cancer risk increased with duration of use of combined hormonal contraceptives and was greater for in situ cancer than for invasive cancer.^10^ Several recent systemic reviews of the link between OC use and cervical cancer indicated that the relative risk for cervical cancer, squamous cell carcinoma, and adenocarcinoma increased with duration of OC use compared with OC never users.^1^^,^^11^^,^^12^

In Korean women, co-factors related to cervical cancer^13^ or to progression to CIN 2 or higher grades with oncogenic HPV infection^14^ include tobacco smoking,^13^ a high number of births (≥3^13^ or ≥4^14^), and marital status (single or married).^14^ In a hospital-based case-control study of 200 women, smoking was associated with CIN (OR 2.49; 95% CI 1.21–5.15) and invasive cervical cancer (OR 3.42; 95% CI 1.59–7.38) compared to ORs in healthy control women, but OC use had no effect on risk.^13^ A retrospective study of 800 oncogenic HPV-infected women with normal or CIN 1 histology demonstrated that neither smoking nor OC use were associated with lesion progression.^14^ These inconsistent findings and the negative association for the risk of cervical neoplasia may be due to the fact that the number of Korean female smokers and OC users is very low. Further, previous studies that included Korean women did not account for the dose-response effect, such as pack-years, the number of cigarettes smoked a day, and duration of OC use, and did not separately assess the risks of CIN 1 and CIN 2 or 3.

A biological interaction between environmental factors, genetic factors, or environmental and genetic factors could lead to either an increase in disease rate or an improvement in health. For example, serum ferritin levels and body mass index have an additive effect on the risk of coronary artery disease.^15^ Cigarette smoking interacts synergistically with chronic infection of hepatitis C virus in men and with heavy alcohol consumption in women.^16^ These reports suggest that smoking in women may interact with OC use in cervical carcinogenesis. Further, a decrease in the number of Langerhans cells was noted in women who smoke,^17^ especially in those using OC, in a study that investigated the association between cotinine levels in the blood and cervical fluid in smokers and non-smokers.^18^ This finding may indicate a synergistic suppression of local cervical immunity by cigarette smoking and OC use.

We hypothesize that cigarette smoking and OC use have a synergistic effect on cervical carcinogenesis that exceeds the risk associated with either factor alone. Therefore, we aimed to assess the biological interaction between cigarette smoking and OC use and between heavy smoking and long-term OC use in the risks of CIN 1 and CIN 2 or 3.

## METHODS

### Study design and population

The HPV cohort study is an ongoing study that includes woman aged 18–65 years who were randomly selected from the gynecologic clinics of 6 university hospitals in Korea and recruited from March 2006 to November 2012. Subjects completed a questionnaire of risk factors for cervical dysplasia or cervical cancer and underwent a physical and gynecological examination. Follow-up visits were planned every 4 months in the first year and every 6 months thereafter. Further details on subject recruitment, including inclusion and exclusion criteria and follow-up procedure, were described in our previous report.^19^ Among 1096 enrolled subjects,^19^ the present report evaluates 678 women who had complete responses to 4 questionnaires regarding tobacco smoking status, secondhand smoking status, oral contraceptive use, and alcohol consumption. The subjects were assigned according to baseline Pap smear pathology to either a control group (with normal or atypical squamous cells of undetermined significance [ASCUS]; *n* = 411), a CIN 1 case group (*n* = 133), or a CIN 2 or 3 case group (*n* = 134). This study was approved by the Institutional Review Board and ethics committee of the Korean National Cancer Center (NCCNCS-06002) and of the Korea University Guro Hospital. All subjects provided written informed consent prior to participation in this study.

### Questionnaire related to co-factors

At the time of enrollment, a lifestyle questionnaire was administered to each subject to obtain a range of medical information that included their height, weight, reproductive and menstrual history, oral contraceptive use history, medical history, and family history of cancer. The sociodemographic characteristics collected for the subjects included level of education, tobacco smoking habits, secondhand smoke exposure, and alcohol consumption. Questions on tobacco smoking, secondhand exposure, OC use, and alcohol consumption included information on smoking status (never, ex-, or current smoker), the current and past numbers of cigarettes usually smoked per day, household exposure to secondhand smoke by partner’s smoking habits or anyone in the house (never, ex-, or current smoker), OC use (never, ex-, or current user) for more than 1 month, period of current or past contraception use (month or year), and alcohol consumption (never, ex-, or current drinker).

### HPV DNA detection, Pap smears, and histological diagnosis

Oncogenic HPV DNA detection was performed using the commercially available Hybrid capture II system (HC-II, Digene Co., Silver Spring, MD, USA). Chemiluminescent HPV DNA tests were measured in relative light units (RLUs) with a probe specific for 13 types of high-risk (HR) HPV (Types 16, 18, 31, 33, 35, 39, 45, 51, 52, 56, 58, 59, and 68). The test results were read as positive at concentrations of 1 pg/mL or greater than the RLU/cutoff ratio (RLUs of specimen/mean RLUs of two positive controls). The cytological grades for the Pap smear reports were based on the Bethesda classification system.^20^ The histological diagnosis was made based on Ki-67 immunostaining.^21^

### Statistical analysis

Chi-squared tests and ANOVA containing post-hoc analyses conducted using the Tukey method were used to analyze differences in the distributions of categorical and continuous variables. A multinomial logistic regression model was used to estimate odds ratios (ORs) and corresponding 95% confidence intervals (CIs) of smoking, OC use, heavy smoking, long-term OC use, combination of smoking and OC use, and combination of heavy smoking and long-term OC use for the risks of CIN 1 and CIN 2/3 (control vs CIN 1 vs CIN 2/3). Unconditional logistic regression analysis was used to estimate the ORs and 95% CIs of smoking, OC use, heavy smoking, long-term OC use, combination of smoking and OC use, and combination of heavy smoking and long-term OC use for the risk of CIN—meaning any of CIN 1, 2, or 3 (control vs CINs).

Combination groups in the additive scaled model were 1) non-smoker and non-OC user, 2) smoker and non-OC user, 3) non-smoker and OC user, and 4) smoker and OC user. Heavy smoking was defined as smoking more than 8 cigarettes per day (the mean number of cigarettes smoked per day in this study), and long-term OC use was defined as OC use for more than 20 months (the mean period of OC use in this study). The risks of smokers without OC use, OC users without smoking, and OC users with smoking were estimated by using non-smokers without OC use as a reference. The *P* value for a linear trend of ORs in the additive scaled model and the *P* value for ORs on a multiplicative interaction were also calculated. Biological interactions in the additive scaled model were validated using the relative excess risk due to interaction (RERI) and synergy index (S), as described by Rothman et al.^22^ RERI, a measure of additive interaction, refers to the excess risk due to interaction compared with the risk without exposure. RERI > 0 means a positive interaction or more than additive effect. The RERI is defined as RR_11_ − RR_10_ − RR_01_ + 1, and SI is defined as [RR_11_ − 1]/[(RR_10_ − 1) + (RR_01_ − 1)].^23^ RERI > 0 and S > 1 indicates a synergistic effect between two cofactors. The Mantel-Haenszel homogeneity test was performed to analyze modification of the effect of OC use on the risk of cancer by smoking status. All logistic regression analyses were adjusted for age, BMI, marital status, menopausal status, alcohol consumption status, and oncogenic HPV infection, which were differentially distributed as categorical variables between the control group and CIN groups. The statistical analysis was performed using the STATA 12.0 (Stata Corp., College Station, TX, USA) software package.

## RESULTS

### General characteristics of the study subjects

The control subjects were older than the subjects with CIN 1 and CIN 2/3 lesions (*P* < 0.001), and the mean BMI of control subjects was slightly higher than that of CIN 1 and CIN 2/3 subjects (*P* = 0.036) (Table 1). The proportions of single and postmenopausal women were higher in the control group than in the CIN 1 or CIN 2/3 groups (*P* < 0.001). The CIN groups also included a higher proportion of tobacco smokers (*P* = 0.004), alcohol drinkers (*P* < 0.001), and HR-HPV-positive subjects (*P* < 0.001) than the control group. The subjects included in this analysis (*n* = 678) were a little older, fatter, less educated, more likely to be married, and slightly less likely to be premenopausal, alcohol drinkers, or HR-HPV infected than the excluded subjects (*n* = 508) (eTable 1).

**Table 1.** General characteristics of study subjects (*n* = 678)

Characteristics	Control	CIN 1	CIN 2 or 3	*P* value^a^
*n*	411	133	134	
Age, years	44.8 (10.2)^b^	39.0 (11.0)^c^	40.9 (10.5)^c^	<0.001
<35	69 (16.8)	52 (39.1)	40 (29.9)	<0.001
35–44	129 (31.4)	38 (28.6)	46 (34.3)	
45–54	145 (35.3)	31 (23.3)	33 (24.6)	
≥55	68 (16.5)	12 (9.0)	15 (11.2)	
Body mass index, kg/m^2^	22.6 (2.9)	22.0 (3.0)	22.0 (3.4)	0.036
<18.5	20 (4.9)	16 (12.0)	15 (11.2)	0.031
18.5–22.9	228 (55.5)	72 (54.1)	75 (56.0)	
23.0–24.9	81 (19.8)	18 (13.6)	25 (18.6)	
≥25	81 (19.8)	27 (20.3)	19 (14.2)	
Education level				
Middle school or less	108 (26.3)	25 (18.8)	40 (29.9)	0.123
High school	175 (42.7)	58 (43.6)	61 (45.5)	
University or more	127 (31.0)	50 (37.6)	33 (24.6)	
Marital status				
Single	26 (6.3)	28 (21.1)	19 (14.2)	<0.001
Married	385 (93.7)	105 (78.9)	115 (85.8)	
Menopausal status				
Pre-menopause	257 (62.7)	109 (82.6)	108 (80.6)	<0.001
Post-menopause	153 (37.3)	23 (17.4)	26 (19.4)	
Number of children				
None or one	56 (16.0)	11 (11.9)	15 (14.5)	0.881
Two	213 (60.9)	59 (64.1)	66 (64.1)	
Three or more	81 (23.1)	22 (24.0)	22 (21.4)	
Oral contraceptive use				
Non-users	350 (85.2)	108 (81.2)	102 (76.1)	0.051
Users^d^	61 (14.8)	25 (18.8)	32 (23.9)	
Tobacco smoking				
Non-smokers	370 (90.0)	107 (80.4)	110 (82.1)	0.004
Smokers^d^	41 (10.0)	26 (19.6)	24 (17.9)	
Secondhand smoking				
Non-smoker	250 (60.8)	70 (52.6)	68 (50.8)	0.060
Smokers	161 (39.2)	63 (47.4)	66 (49.2)	
Alcohol consumption				
Non-drinkers	207 (50.4)	39 (29.3)	48 (35.8)	<0.001
Drinkers^d^	204 (49.6)	94 (70.7)	86 (67.2)	
HR-HPV DNA^e^				
Negative	296 (72.2)	19 (18.8)	32 (38.6)	<0.001
Positive	114 (27.8)	82 (81.2)	51 (61.4)	

CIN, cervical intraepithelial neoplasia; HR-HPV, high-risk human papillomavirus.

Only the variables available were used in this study, as not all 678 women completed all questionnaires fully.

Continuous variables are presented as mean (SD) and categorical variables are presented as number (%).

^a^Distributional differences of continuous and categorical variables were confirmed by using ANOVA and chi square tests, respectively.

^b,c^Different letters indicate that means between two subject categories are significantly different (Tukey HSD, *P* < 0.05).

^d^Includes both current and past status.

^e^HR-HPV DNA was detected using a Hybrid Capture II assay for detecting 13 oncogenic HPV DNA types.

### Single effect of co-factors on the risk of CIN

After adjustment for all potential confounding factors in this study, OC use had no effect on the risk of CIN 1 or CINs, but was associated with an increased risk of CIN 2/3 (OR 1.98; 95% CI, 1.07–3.69) compared with the no use group (Table 2). Long-term OC use (≥20 months) was not associated with risk of CIN 1 or CINs, but was associated with an increased risk of CIN 2/3 (OR 2.71; 95% CI, 1.11–6.59) compared with non-OC use. Neither being a smoker nor smoking more than 8 cigarettes a day had a significant effect on the risk of any CIN grade. Secondhand smoking was also not associated with risk of any CIN grade (eTable 2).

**Table 2.** Odds ratios of oral contraceptive use and smoking for cervical intraepithelial neoplasia risk

Status	CIN 1 (*n* = 133)		CIN 2 or 3 (*n* = 134)		CINs (*n* = 267)	
		
	Case/Control	OR (95% CI)^b^	Case/Control	OR (95% CI)	Case/Control	OR (95% CI)
OC use^a^						
Non-users	108/350	1 (ref.)	102/350	1 (ref.)	210/350	1 (ref.)
Users (current/past)	25/61	1.11 (0.57–2.15)	32/61	1.98 (1.07–3.69)	57/61	1.51 (0.89–2.56)
Duration of OC use						
No use	108/350	1 (ref.)	102/380	1 (ref.)	210/350	1 (ref.)
Less than 20 months	17/38	1.08 (0.50–2.36)	15/38	1.63 (0.76–3.49)	32/38	1.33 (0.70–2.53)
20 months or more	8/23	1.12 (0.39–3.21)	17/23	2.71 (1.11–6.59)	25/23	1.86 (0.84–4.12)
Tobacco smoking^a^						
Non-smokers	107/370	1 (ref.)	110/370	1 (ref.)	217/370	1 (ref.)
Smokes	26/41	1.75 (0.78–3.90)	24/41	1.56 (0.72–3.41)	50/41	1.65 (0.86–3.16)
Cigarettes smoked per day						
Non-smoker	107/370	1 (ref.)	110/370	1 (ref.)	217/370	1 (ref.)
Less than 8 cigarettes/day	17/21	2.39 (0.94–6.07)	9/21	1.23 (0.43–3.50)	26/21	1.76 (0.80–3.89)
8 cigarettes/day or more	9/20	0.98 (0.29–3.33)	15/20	1.92 (0.43–3.50)	24/20	1.51 (0.61–3.75)

CI, confidence interval; CIN, cervical intraepithelial neoplasia; OC, oral contraceptive; OR, odds ratio.

Subjects with normal or atypical squamous cells of undetermined significance in Pap test were included in the control group (*n* = 411), and subjects with CIN 1, CIN 2, and CIN 3 on biopsy were included in CINs group.

^a^Includes both current and past status.

^b^Multinomial logistic regression analysis (Control vs CIN 1 vs CIN 2 or 3) and unconditional logistic regression analysis (Control vs CINs) were performed after adjustment for age, body mass index, marital status, menopausal status, alcohol consumption status, and oncogenic human papillomavirus infection as categorical variables.

### Joint effect of co-factors on CIN risk

A joint effect model was designed based on an additive scale of smoking and OC use and used to evaluate the biological interaction (Table 3 and Table 4). Subjects who smoked and used OCs had a higher risk of CIN 2/3 (OR 4.91; 95% CI, 1.68–14.4; RERI/S, 3.77/27.4; *P* for multiplicative interaction = 0.003) and CINs (OR 3.81; 95% CI, 1.45–10.1; RERI/S, 2.39/6.64; *P* for multiplicative interaction = 0.009) than those who did not smoke or use OCs (Table 3). The *P* values for homogeneity test of OC use after stratification by smoking status were 0.025 in CIN 2/3 and 0.038 in CINs. However, no significant biological interaction from this joint exposure was observed in the CIN 1 group. Heavy smoking (≥8 cigarettes/day) and long-term OC use (≥20 months) also increased risk of CIN 2/3 (OR 11.5; 95% CI, 1.88–70.4; RERI/S, 9.93/18.8; *P* for multiplicative interaction = 0.009) and CINs (OR 6.73; 95% CI, 1.13–40.1; RERI/S, 5.32/15.6; *P* for multiplicative interaction = 0.038) versus not smoking smoke or not using OCs. The *P* values for homogeneity test of long-term OC use after stratification by heavy smoking were 0.095 in CIN 2/3 risk and 0.082 in CINs risk. The combination of secondhand smoking and OC had no effect on increase of any CIN risk (eTable 2).

**Table 3.** Odds ratios of combination of oral contraceptive use and smoking status for cervical intraepithelial neoplasia

Status	CIN 1		CIN 2 or 3		CINs	
		
	Case/Control	OR (95% CI)^a^	Case/Control	OR (95% CI)	Case/Control	OR (95% CI)
Non-smoker & Non-OC user	92/318	1 (ref.)	92/318	1 (ref.)	184/318	1 (ref.)
Non-smoker & OC user	15/52	1.05 (0.47–2.33)	18/52	1.28 (0.61–2.71)	33/52	1.21 (0.66–2.24)
Smoker & Non-OC user	16/32	1.71 (0.65–4.53)	10/32	0.86 (0.28–2.62)	26/32	1.21 (0.55–2.66)
Smoker & OC user	10/9	2.42 (0.65–8.98)	14/9	4.91 (1.68–14.4)	24/9	3.81 (1.45–10.1)
RERI/S^b^		0.66/1.87		3.77/27.4		2.39/6.64
*P* for interaction^c^		0.258		0.003		0.009
*P* for homogeneity^d^		0.208		0.025		0.038
Non-smoker & Non-OC user	92/318	1 (ref.)	92/318	1 (ref.)	184/318	1 (ref.)
≥8 cigarettes/day & Non-OC user	5/14	0.91 (0.14–6.09)	7/20	1.67 (0.38–7.27)	11/20	1.14 (0.38–3.38)
Non-smoker & OC use for ≥20 months	4/20	1.15 (0.14–6.09)	6/14	0.89 (0.22–3.56)	11/14	1.23 (0.34–4.47)
≥8 cigarettes/day & OC use for ≥20 months	2/2	2.34 (0.11–49.7)	6/2	11.5 (1.88–70.4)	8/2	6.73 (1.13–40.1)
RERI/S		1.28/20.9		9.93/18.8		5.32/15.6
*P* for interaction		0.581		0.009		0.038
*P* for homogeneity		0.266		0.095		0.082

CI, confidence interval; CIN, cervical intraepithelial neoplasia; OC, oral contraceptive; OR, odds ratio; RERI, relative excess risk due to interaction; S, synergy index.

^a^Multivariate odds ratio was calculated using the non-smoker and non-OC-user group as a reference after adjustment for age, body mass index, marital status, menopausal status, alcohol consumption status, and oncogenic human papillomavirus infection as categorical variables.

^b^RERI and S of the additive scaled model were calculated as described by Rothman et al. RERI > 0 and S > 1 indicates a synergistic effect between two factors.

^c^The *P* is for interaction of the multiplicative term (smoking status × OC use, heavy smoking × long-term OC use).

^d^Test of homogeneity using the Mantel-Haenszel method was performed after stratification of smoking and oral contraceptive use. (Hypothesis: The effects of oral contraceptive use on the risk of CINs in non-smokers and smokers are equal.)

**Table 4.** Odds ratios of combination of oral contraceptive use and smoking status for cervical intraepithelial neoplasia when stratified as two groups of <35 and ≥35 years of age

Status	CIN 1		CIN 2 or 3		CINs	
		
	Case/Control	OR (95% CI)^a^	Case/Control	OR (95% CI)	Case/Control	OR (95% CI)
*≤34 years of age*						
Non-smoker & Non-OC user	33/47	1 (ref.)	20/47	1 (ref.)	53/47	1 (ref.)
Smoker & Non-OC user	8/11	0.79 (0.17–3.67)	7/11	1.72 (0.37–7.93)	15/11	1.09 (0.34–3.53)
Non-smoker & OC user	4/6	0.54 (0.09–3.17)	4/6	2.07 (0.42–10.1)	8/6	1.12 (0.28–4.48)
Smoker & OC user	7/5	1.29 (0.23–7.33)	9/5	6.52 (1.39–30.7)	16/5	3.42 (0.91–12.8)
*P* for interaction^b^		0.619		0.028		0.066
*≥35 years of age*						
Non-smoker & Non-OC user	59/271	1 (ref.)	72/271	1 (ref.)	131/271	1 (ref.)
Smoker & Non-OC user	8/21	2.47 (0.69–8.86)	3/21	0.33 (0.04–2.84)	11/21	1.17 (0.39–3.51)
Non-smoker & OC user	11/46	1.25 (0.51–3.08)	14/46	1.19 (0.50–2.84)	25/46	1.27 (0.64–2.53)
Smoker & OC user	3/4	5.56 (0.75–41.1)	5/4	5.37 (1.09–26.4)	8/4	4.87 (1.16–20.4)
*P* for interaction		0.114		0.030		0.034

CI, confidence interval; CIN, cervical intraepithelial neoplasia; OC, oral contraceptive; OR, odds ratio.

^a^Multivariate odds ratio was calculated using non-smoker and non-oral contraceptive user as a reference after adjustment for age, body mass index, marital status, menopausal status, alcohol consumption status, and oncogenic human papillomavirus infection as categorical variables.

^b^The *P* is for interaction of the multiplicative term (smoking status × OC use).

### Joint effect of co-factors on CIN risk in subjects <35 and ≥35 years of age

Age was distributed differentially between the groups in this study. We separately examined the association of smoking and OC use joint with CIN in subjects less than 35 years of age and in subjects 35 years of age and older (Table 4). There was no significant association between each co-factor and the risk of CIN in either subjects <35 or ≥35 years of age. In subjects <35 years of age, subjects who smoked and used OCs had a higher risk of CIN 2/3 (OR 6.52; 95% CI, 1.39–30.7; *P* for multiplicative interaction = 0.028) and CINs (OR 3.42; 95% CI, 0.91–12.8; *P* for multiplicative interaction = 0.066) than those who did not smoke and did not use OCs. In subjects aged ≥35 years, subjects who smoked and used OCs had a higher risk of CIN 2/3 (OR 5.37; 95% CI, 1.09–26.4; *P* for multiplicative interaction = 0.030) and CINs (OR 4.87; 95% CI, 1.16–20.4; *P* for multiplicative interaction = 0.034) than those who did not smoke or use OCs.

## DISCUSSION

We demonstrated that any OC use and long-term OC use (≥20 months) was associated with elevated risk of CIN 2/3 but not with CIN 1. Cigarette smoker and heavy smoking (≥8 cigarettes/day) had no association with either CIN 1 or CIN 2/3. Smokers with OC use and heavy smoking with long-term OC use showed a marked synergistically increased CIN 2/3 risk, but this effect was not observed for CIN 1 risk. This synergistic joint effect was apparent in both two age groups (<35 and ≥35 years of age).

A systematic review provided potential evidence regarding the correlation of OC use with squamous cells carcinoma.^24^ The RR of cervical cancer increased with the duration of OC use (RRs of 1.1 and 2.2 for <5 years and >10 years, respectively). The results were similar to those for invasive carcinoma and CIN 3 as well as for squamous cell carcinoma and adenocarcinoma. According to the cumulative results of 24 epidemiological studies, the RR of invasive cervical cancer increased with duration of OC use and decreased after discontinuation of OC use.^25^ This study also showed a positive association of ever OC use and long-term OC use with CIN 2/3 risk. However, the association with OC use with risk was not observed in previous studies of Korean women,^13^^,^^14^ possibly because duration of OC use was poorly considered and the association was not assessed by CIN grade. For studies on OC use, a dose-response effect should be taken into account, such as years of OC use.^26^

Smoking has been regarded as the most significant environmental risk factor for cervical cancer, and the risk increases with the intensity and duration of smoking.^11^ Smoking was associated with cervical cancer among HPV-positive Korean women in one study,^13^ but another study reported no association between smoking and progression to severe lesions.^14^ Smoking among Korean women is rare (6.8% of female ≥19 years of age were smokers in 2011).^27^ Because the proportion of current smokers (6.3% in control group) was very low in this study, both current and former smokers were included in the smoker group. The rate of smokers in this study (10.1%) was similar to that in the previous study (10.7%, current and former smokers).^13^ Even with the inclusion of current and former smokers, no effect of smoking on CIN risk was observed. However, this result may be due to the dilution of current smoking’s effect by the inclusion of former smokers, so these results should be validated using a larger number of current female smokers. In contrast to the percentage of female smokers, the percentage of Korean men who smoke is high (47.3% of males aged ≥19 years were smokers in 2011). Similar tobacco use patterns are found in other Asian countries, where studies have demonstrated a possible effect of exposure to secondhand smoke on the risk of cervical neoplasia.^28^^–^^30^ Although secondhand smoke was not associated with CINs in this study, the effect of secondhand smoke should be assessed using more detailed questionnaires.

Steroid hormones may increase transcription of the HPV E6 oncogene, leading to degradation of the p53 gene product and failure of G1/S cell-cycle arrest, thereby inducing carcinogenesis.^31^ A direct oncogenic effect of benzopyrene, a chemical carcinogen from tobacco, is enhanced HPV synthesis of cervical cells,^32^ which might lead to viral persistence. Genome amplification could result in increased copies of oncogenes E6 and E7. Smoking causes inactivation of glutathione S-transferases, detoxifying an activated form of the carcinogen in epithelial tumor cells.^33^^,^^34^ Cell-mediated immunity against HPV infection was also suppressed by smoking.^17^^,^^35^ Smoking has been correlated with a decrease in numbers of Langerhans cells and helper/inducer T lymphocytes in the squamous epithelial transformational zone of the cervix.^17^

Our results suggest that a combination of cigarette smoking and OC use could strongly influence the development of high-grade CIN lesions. This phenomenon has been described in several other research reports. In one study, in which DNA adducts in the human cervix were measured using ^32^P-postlabeling assays, a significant difference was found between the DNA adduct levels obtained from the cervical DNA of smokers who had used OCs and those who had not.^36^ Another study showed that aneuploidy, serum progesterone concentrations, and habitual smoking were significantly associated with the fraction of DNA in the S-phase (a marker of tumor growth) in squamous cervical cancer.^37^ In a study that investigated the association between cotinine levels in the blood and cervical fluid of smokers and non-smokers, a decrease in the number of Langerhans cells was noted in smokers, especially in those using OCs, and in the densities of Langerhans cells or macrophages in normal uterine cervices.^18^ Langerhans cells may be the initial cellular targets in the sexual transmission of HIV. Langerin, a protein found in Langerhans cells, is able to scavenge viruses from the surrounding environment, thereby preventing infection.^38^ Taken together, the joint effects of smoking and OC use may result in synergistic suppression of local cervical immunity. This should be supported by measurements of the immune cell numbers in the cervix.

Interestingly, combined cigarette smoking and OC use was associated with more severe CIN 2 or 3 lesions but not with the milder CIN 1 lesions or current HR HPV infection. The importance of histological differentiation of CIN 1 from CIN 2 or 3 cervical precancerous lesions has been suggested.^39^ A 2-year follow-up study reported that the cumulative risk of CIN 2 or 3 was equivalent for low-grade squamous intraepithelial lesions (LSILs) and HPV-positive ASCUS.^40^ This means that the risk is not significantly different between CIN 1 at initial histological distinction and negative colposcopy and biopsy. It has been reported that distinct covariates of HR HPV are associated with progression to CIN 1, CIN 2, and CIN 3.^41^ Luhn et al reported that long-term OC use, multiparity, smoking, and not having a Pap test were associated with CIN 3 compared to CIN 2.^42^ This suggests that hormone-related cofactors and smoking may play an important role at the transition from HPV infection to cervical precancer. Further, in a previous study, we showed that alcohol consumption and viral load are synergistically associated only with CIN 1, not CIN 2/3 or cervical cancer.^43^ Taken together, these findings suggest that co-factors acting in each stage of cervical carcinogenesis may vary; in this population, alcohol and viral load can play a role in oncogenic HPV infection or its persistence and the transition to CIN 1, while OC use and combined OC use with cigarette smoking can contribute to the transition to precancerous CIN 2 or 3 lesions.

Although this study detected a clear interaction between smoking and OC use, the sample size for interaction analysis in a case-control study should be much higher than that used in the original analysis. This may account for the broad CIs that were calculated using the joint effect model. In addition, details derived from questionnaires for each cofactor regarding the frequency, amount, duration, and exposure time to each cofactor were not used sufficiently in this analysis. As such, some useful variables, such as pack-years of smoking, were not included in this study, which may have resulted in overestimation or underestimation of the degree of interaction. The case-control design resulted in various limitations, such as recall bias, selection bias, examination of a single outcome, inability to estimate incidence rates of disease, and difficulty in determining the temporal sequence between exposure and disease.^44^ Finally, our study is limited by the lack of information on sexual behavior or disease transmission, a confounding factor for HPV infection. Although a reduced frequency of sexual intercourse and an inactive sexual life have been reported among middle-aged Korean women,^45^ sexual behaviors might affect the association.

In conclusion, we suggest that long-term OC use may be associated with cervical precancerous lesions, and the combined smoking and OC use should be regarded as a major risk factor for severe cervical dysplasia. Clinicians are therefore recommended to encourage women with normal or low-grade dysplasia to avoid combining smoking and OC use in order to prevent progression of severe cervical dysplasia. Additional studies that include a larger sample size and molecular approaches are required to confirm this interaction and elucidate the underlying mechanisms.

## ONLINE ONLY MATERIALS

eTable 1. General characteristics of the subjects included and not included in this study.

eTable 2. Odds ratio of combination of secondhand smoking status and oral contraceptive use for cervical intraepithelial neoplasia.
