# Supplementary material for: Association of Combined Tobacco Smoking and Oral Contraceptive Use With Cervical Intraepithelial Neoplasia 2 or 3 in Korean Women
Source: J Epidemiol. 2016 Jan 5;26(1):22–9. doi: 10.2188/jea.JE20150047 (PMC4690737; doi:10.2188/jea.JE20150047)
Supplement: eTable 1. [file je-26-022-s001.pdf]

eTable 1. General characteristics of the subjects included and not included in this study

Characteristics	Subjects included	Subjects not included	*p* value[a]
n	678	508	
Age, years	42.8 (10.7)	41.4 (10.9)	0.036
Body mass index, kg/m^2	22.4 (3.0)	21.7 (2.6)	0.001
Highest education level			
Middle school or less	25.6	15.6	< 0.001
High school	43.4	44.2	
University or more	31.0	40.2	
Marital status			
Single	10.8	16.8	0.004
Married	89.2	83.2	
Menopausal status			
Pre-menopause	70.1	77.4	0.009
Post-menopause	29.9	22.6	
Tobacco smoking			
Non-smokers	86.6	83.1	0.116
Smokers	13.4	16.9	
Secondhand smoking			
Non-smokers	57.2	55.9	0.665
Smokers	42.8	44.1	
Alcohol consumption			
Non-drinkers	43.4	27.9	< 0.001
Drinkers	56.6	72.1	
HR-HPV DNA[b]			
Negative	58.4	45.1	< 0.001
Positive	41.6	54.9	

HR-HPV, high-risk human papillomavirus.

Among 1,096 subjects enrolled in this cohort, only 678 subjects with information on smoking, oral contraceptive use, and secondhand smoking were included in this study.

Continuous variables are presented as mean (standard deviation), and categorical variables are presented as n (%).

[a] Distributional differences of continuous and categorical variables were confirmed using the t-test and chi square test, respectively.

[b] HR-HPV DNA was detected using a Hybrid Capture II assay for detecting 13 oncogenic HPV DNA types.
