# Supplementary material for: Association of Combined Tobacco Smoking and Oral Contraceptive Use With Cervical Intraepithelial Neoplasia 2 or 3 in Korean Women
Source: J Epidemiol. 2016 Jan 5;26(1):22–9. doi: 10.2188/jea.JE20150047 (PMC4690737; doi:10.2188/jea.JE20150047)
Supplement: eTable 2. [file je-26-022-s002.pdf]

eTable 2. Odds ratio of combination of secondhand smoking status and oral contraceptive use for cervical intraepithelial neoplasia

Status	CIN 1		CIN 2 or 3		CINs	
	Case/Control	OR (95% CI)[a]	Case/Control	OR (95% CI)	Case/Control	OR (95% CI)
Secondhand smoking						
Non-SHS	70/250	1 (ref.)	68/250	1 (ref.)	138/250	1 (ref.)
SHS	63/161	1.13 (0.66–1.95)	66/161	1.31 (0.76–2.27)	129/161	1.21 (0.80–1.85)
Non-SHS & Non-OC user	57/214	1 (ref.)	50/214	1 (ref.)	107/214	1 (ref.)
SHS & Non-OC user	51/136	1.26 (0.70–2.28)	118/36	1.44 (0.78–2.67)	31/36	1.31 (0.82–2.09)
Non-SHS & OC user	13/36	1.48 (0.59–3.70)	52/136	2.24 (0.98–5.10)	103/136	1.83 (0.92–3.65)
SHS & OC user	12/25	1.03 (0.37–2.88)	14/25	2.11 (0.78–5.75)	26/25	1.58 (0.70–3.56)

CI, confidence interval; CIN, cervical intraepithelial neoplasia; OC, oral contraceptive; OR, odds ratio; SHS, secondhand smoking.

[a] Multivariate odds ratio was calculated by using the non-secondhand smoker and non-OC user group as a reference after adjustment for age, body mass index, marital status, menopausal status, smoking status, alcohol consumption status, and oncogenic human papillomavirus infection as categorical variables.
